# Emotional Processing, Recognition, Empathy and Evoked Facial Expression in Eating Disorders: An Experimental Study to Map Deficits in Social Cognition

**DOI:** 10.1371/journal.pone.0133827

**Published:** 2015-08-07

**Authors:** Valentina Cardi, Freya Corfield, Jenni Leppanen, Charlotte Rhind, Stephanie Deriziotis, Alexandra Hadjimichalis, Rebecca Hibbs, Nadia Micali, Janet Treasure

**Affiliations:** 1 Section of Eating Disorders, Psychological Medicine, King’s College London, Institute of Psychiatry, London, United Kingdom; 2 Behavioural and Brain Sciences Unit, Institute of Child Health, University College London, London, United Kingdom; Bournemouth University, UNITED KINGDOM

## Abstract

**Background:**

Difficulties in social cognition have been identified in eating disorders (EDs), but the exact profile of these abnormalities is unclear. The aim of this study is to examine distinct processes of social-cognition in this patient group, including attentional processing and recognition, empathic reaction and evoked facial expression in response to discrete vignettes of others displaying positive (i.e. happiness) or negative (i.e. sadness and anger) emotions.

**Method:**

One hundred and thirty-eight female participants were included in the study: 73 healthy controls (HCs) and 65 individuals with an ED (49 with Anorexia Nervosa and 16 with Bulimia Nervosa). Self-report and behavioural measures were used.

**Results:**

Participants with EDs did not display specific abnormalities in emotional processing, recognition and empathic response to others’ basic discrete emotions. However, they had poorer facial expressivity and a tendency to turn away from emotional displays.

**Conclusion:**

Treatments focusing on the development of non-verbal emotional communication skills might be of benefit for patients with EDs.

## Introduction

Human relationships are regulated by cognitive processes, such as perception, attention, memory and motivation, used to understand and respond to social stimuli. These processes have been identified as “social cognition” and include skills of varying degrees of complexity, such as recognising and attributing meaning to self and others’ emotions, thoughts and intentions [1, 2]. Most psychiatric disorders are characterised by impairments in social functioning, which have been investigated by exploring the underlying abnormalities in social cognition. Autism spectrum disorders (ASDs) are a prototypical example of primary disorders of social cognition, with deficits consistently identified in tracking others’ mental states from early childhood [3]. The development of complex interpersonal relationships and challenges during adolescence triggers the onset of social difficulties in disorders such as social anxiety and substance abuse. However, in these conditions it seems less clear whether social difficulties are due to primary disorders of social cognition or are secondary to other problems [3]. Inconsistent results have been found in some psychiatric disorders and in part this arises from the use of different assessment methods probing various complex facets of social cognition [4].

One approach to the study of social cognition is to build evidence from paradigms that focus on discrete emotions and clearly defined basic processes [5]. Happe and Frith [3] have mapped the different processes and aspects that characterise social cognition according to distinct underlying neural substrates. This model includes the constructs of agent identification (e.g. voice and face processing and eye gaze tracking), emotion processing (e.g. recognition of emotional expressions), empathy (i.e. own emotional response to others’ emotional expressions), self-processing (i.e. self-awareness of own emotional and bodily state) and social hierarchy (e.g. stereotyping of social groups with links to affiliation and mimicry) [3].

Severe impairments in social functioning characterise patients with eating disorders (EDs [6–8]) and have a detrimental impact on treatment outcomes and prognosis [8]. The facets of social-cognition that appear to be compromised in this patient group include a biased perception of emotional stimuli (e.g. attentional bias to threatening faces [9, 10]), difficulties identifying basic and complex emotions [11, 12] and displaying emotional empathy [13, 14], and reduced facial emotional expressivity [15, 16]. However, findings from these studies are inconsistent and/or difficult to interpret because of limitations. Attentional biases towards social stimuli have been investigated only in response to complex emotional expressions (i.e. rejecting/accepting faces; dominant/submissive faces [9, 10]). Similarly, deficits in emotion recognition have been mainly identified during complex emotion recognition tasks or tasks involving paradigms with limited ecological validity (i.e. forced-choices in response to standard emotional faces or eyes [11, 12]). Empathy has been measured with self report instruments [13, 14]. Evoked facial emotional expression has been measured in response to brief scenes from popular movies [15, 16] rather than using more basic emotional paradigms.

The aim of this study was to experimentally investigate specific processes of social-cognition based on Happe and Frith’s map [3] to identify the constructs of agent identification (i.e. faces processing), emotion processing (i.e. recognition of others’ emotional expressions), empathy (i.e. own emotional response to others’ emotional expressions) and evoked facial emotional expressions in patients with anorexia nervosa or bulimia nervosa. These processes were assessed in response to others’ displays of positive (i.e. happiness) and negative (i.e. sadness and anger) distinct, basic emotions due to the differential impact of these emotions on behaviour [17, 18] and the paucity of studies investigating basic, rather than complex emotions.

### Hypotheses

We hypothesised that women with an ED compared to healthy controls (HCs) would: 1) selectively attend to expressions of sadness and disengage from expressions of happiness during the attentional probe task; 2) show deficits in the recognition of others’ positive or negative emotions in short video clips; 3) report lower emotional empathy in response to the video-clips; 4) have less evoked emotional facial expressivity and a greater tendency to turn away from the video clips depicting both positive or negative emotions.

## Methods

### Ethics statement

The investigation was carried out in accordance with the latest version of the Declaration of Helsinki. The study received ethical approval from King’s College London (PNM/10/11-111) and all participants provided written informed consent after the nature of the procedures had been fully explained. No minors/children were included in the study.

### Participants

One hundred and thirty-eight female participants were included in the study: 73 healthy controls (HCs) and 65 individuals with an ED. Of those with an ED, 49 had Anorexia Nervosa (AN; *N* = 31 inpatients, *N* = 18 outpatients), and 16 individuals had Bulimia Nervosa (BN; *N* = 3 inpatients, *N* = 13 outpatients).

Participants were recruited from the Institute of Psychiatry Eating Disorders Unit’s volunteer database, through advertisements placed on the BEAT (Beating Eating Disorders) website and through a circular email sent out to the staff and students at King's College London and University College London (UCL). Participants with an ED were also recruited from 2 inpatient units. Inclusion criteria were: women between 16 and 55 years old, fluent in English, with normal visual acuity and no motor impairment. A tailored version of SCID-I (only the overview, screening and EDs modules, and open questions on past or present history of anxiety and mood disorders), which is a standardised interview for diagnostic assessment of DSM-IV disorders [19], was administered to screen for past or current mental health disorder in HCs and to confirm the diagnosis of EDs.

### Measures

A demographic questionnaire including questions on: ethnicity, medication, visual impairment, neurological condition, employment status, current occupation, years of education, eating disorders duration, highest/current/lowest body mass index (BMI), marital status, number of children, household sharing, diagnosis of psychiatric conditions in the family and comorbidity was completed by all participants (Table 1). Participants also completed the following measures:

**Table 1:** Socio-demographic and clinical variables. Socio-demographic and clinical variables compared between groups, expressed as mean (standard deviation), and percentage. One-way ANOVAs followed by posthoc analyses, independent t-tests, and Chi-square tests calculated. Anorexia Nervosa restrictive subtype = AN r, Anorexia Nervosa binge purge subtype AN b/p, Bulimia Nervosa = BN; Healthy Controls = HCs; NS = non significant.

	AN r (N = 19) AN b/p (N = 14)	BN (N = 12)	HCs (N = 71)	Test statistic
Age	28.2 (10)	23.4(5.7)	26.4 (7.8)	F (2,112) = 1.5, p = NS
Years of education	16.3(2.8)	15.9 (2.3)	17.7 (2.9)	F (2,108) = 3.6, p = .03; AN vs. HCs: p = .07; AN vs. BN: p = NS; BN vs. HCs: p = NS
Body Mass Index (Kg/m^2^)	15.9 (1.8)	21.8 (2.3)	21.9 (2.8)	F (2,105) = 72.3, p < .0001; AN vs. HCs: p < .0001; AN vs. BN: p < .0001; BN vs. HCs: p = NS
Length of illness (months)	58.5 (80.7)	32.2 (44.0)	N/A	t(44) = 1.1, p = NS
Psychiatric medication (yes/no)	79.4%	75%	N/A	X^2^ = .1, p = NS
Previous hospital admissions (yes/no)	66.7%	54.5%	N/A	X^2^ = .5, p = NS
Psychiatric disorder other than ED (yes/no)	38.2%	61.8%	N/A	X^2^ = 2.9, p = .09
Without a partner (single/divorced vs. in a relationship)	70.6%	58.3%	45.1%	X^2^(4) = 10, p = .04; AN vs. BN = NS; AN + BN vs. HCs: X^2^(2) = 7.8, p = .02
EDE-Q Restriction	3.7 (1.7)	3.8 (1.6)	.7 (.9)	F (2,113) = 79.9, p < .0001; AN vs. HCs: p < .0001; AN vs. BN: p = NS; BN vs. HCs: p < .0001
EDE-Q Eating Concern	3.5 (1.2)	3.8 (1.3)	.2 (.4)	F (2,113) = 239, p < .0001; AN vs. HCs: p < .0001; AN vs. BN: p = NS; BN vs. HCs: p < .0001
EDE-Q Weight Concern	3.9 (1.5)	4.7 (1.5)	.8 (.9)	F (2,113) = 107.6, p < .0001; AN vs. HCs: p < .0001; AN vs. BN: p = NS; BN vs. HCs: p < .0001
EDE-Q Shape Concern	4.4 (1.6)	4.9 (1.3)	1.0 (1.0)	F (2,113) = 113.3, p < .0001; AN vs. HCs: p < .0001; AN vs. BN: p = NS; BN vs. HCs: p < .0001
EDE-Q Total	3.9 (1.2)	4.3 (1.3)	.7 (.7)	F (2,113) = 161.8, p < .0001; AN vs. HCs: p < .0001; AN vs. BN: p = NS; BN vs. HCs: p < .0001
DASS Stress	26.7 (10.1)	22.3 (11.8)	6.8 (5.9)	F (2,112) = 75.5, p = < .0001; AN vs. HCs: p < .0001; AN vs. BN: p = NS; BN vs. HCs: p = .002
DASS Depression	23.8 (13.1)	26.5 (12.1)	2.4(3.0)	F (2,112) = 98.2, p < .0001; AN vs. HCs: p < .0001; AN vs. BN: p = NS; BN vs. HCs: p < .0001
DASS Anxiety	15.2 (10.9)	15 (9.2)	2.2 (3.2)	F (2,112) = 47.1, p < .0001; AN vs. HCs: p < .0001; AN vs. BN: p = NS; BN vs. HCs: p = .001
Social Support	10.1 (2.4)	8.1 (1.7)	10.9 (2.0)	F (2,114) = 9.3, p < .0001 AN vs. HCs: p = NS AN vs. BN: p = .008 BN vs. HCs: p < .0001

#### Eating Disorder Examination Questionnaire (EDE-Q [20])

This questionnaire is a 36 item self-report version of the original interview. The EDE-Q is composed of four subscales: weight concern, shape concern, eating concern, dietary restraint and a global score (a composite mean score of the four subscales). Scores ranging from 0 to 6 on a Likert scale correspond to the number of days over the past 4 weeks the respondent had experienced a specific attitude, feeling or behaviour. Previous studies show that the EDE-Q has high internal consistency [20] and moderate to high concurrent and criterion validity [21]. The Cronbach’s alpha for the sample tested in this study was 0.9 for the dietary restraint, eating concern and shape concern subscales and 0.6 for the weight concern subscale.

#### Depression Anxiety Stress Scales (DASS-21 [22])

The DASS is a 21-item three-scale self-report measure of depression, anxiety, and stress. Higher scores are related to a higher level of depression, anxiety and stress. The scale has been validated and found to possess good reliability, with Cronbach’s alpha to be 0.94 for Depression, 0.87 for Anxiety and 0.91 for Stress [23]. The Cronbach’s alpha for the sample tested in this study was 0.9 for all subscales.

#### Oslo Social Support Scale (OSS; [24])

The OSS is a 3-item self-report measure. It assesses perception of general social support. The total score is calculated by adding up the raw scores for each item. The sum of the raw scores has a range from 3–14. A score ranging between 3 and 8 is classified as poor support, a score between 9 and 11 as intermediate support, and a score between 12 and 14 as strong support [25].

#### Attentional probe detection task

This test assesses attentional bias. It is an attentional probe-detection task originally developed by Posner, Snyder and Davidson [26]. The participant's task is to respond to a probe stimulus that is initially hidden from view behind one of two stimuli. A fast reaction time (RT) suggests that attention has been directed to the stimulus that obscured the probe.

The task was programmed using E-Prime Psychology Software (Psychology Software Tools, Inc., Pittsburgh, PA) and presented on a 15inch screen laptop. Participants were tested in one of two different rooms, using the same laptop and instructed to sit at a distance from the screen from which they could conformably read the task’ instructions and identify the stimuli. The experimenter remained in the room while the participant completed the tasks.

The stimuli were 24 photographs of adult faces showing happy, sad, and neutral prototypical expressions. The photographs were obtained with permission from the Pictures of Facial Affect set [27]. Twelve happy-neutral pairs and 12 sad-neutral pairs were repeated twice and presented in random order for each participant, for a total of 16 practice and 64 experimental trials.

Each trial started with a fixation point shown on the computer screen for 500 ms and then replaced by a picture pair which appeared for 500 ms. Immediately after the offset of each picture pair, a probe (either: or..) was presented in the location of one of the pictures. The probe remained on the screen until the participant made a response by pressing the appropriate labelled key on the keyboard. Participants were instructed to indicate, as quickly and accurately as possible, which probe appeared on the screen after the presentation of the picture pair. The experimenter remained in the room whilst the participant completed the task.

#### Film Task

Four video clips were presented to participants on a 15-inch computer screen. Each clip showed one adult displaying a discrete emotion: happiness (joyous and smiling), sadness (upset and crying), anger (annoyed) and neutral. The clips were sourced from YouTube and rated by fifteen HCs. They were selected as depicting with most clarity and intensity the appropriately valenced response on the Emotional Assessment Scale and the Positive and Negative Affect Scale. The clips were matched according to duration and intensity of the display of a single person in the frame. Each film clip was ~ 1 minute in length. The order of the clips was fixed. The positive video-clip was displayed before the negative video-clips due to the potential more lasting effect of negative mood [15]. The neutral video clip was presented at baseline and also after each emotionally valenced video clip in order to neutralise carry over effects [15].

Participants rated the film clips according to: 1) valence and intensity of emotion displayed (i.e. “emotion recognition”: 17 adjectives rated on a 5-point scale, Emotional Assessment Scale—EAS; [28]); and 2) valence and intensity of emotion experienced whilst watching the video-clip (“empathy”: 22 adjectives rated on a 5-point scale, Positive and Negative Affect Scale- PANAS [29]). A positive total score and a negative total score were derived for “emotion recognition” and “empathy”. Higher scores represented more intense positive/negative emotions identified/experienced.

### Procedure

This study was carried out in a single 90-min. session. The SCID-I was administered at the beginning of the session, followed by the questionnaires, the attentional probe task, and the clips task. During the clips, participant’s faces were recorded with their consent using a small video camera on a tripod behind the screen. After watching each film clip participants completed the Emotional Assessment Scale (EAS) and the Positive and Negative Affect Scale (PANAS). At the end of the session, height and weight measures were obtained by the experimenter in order to calculate the BMI (Kg/m^2^).

### Statistical analyses

Statistical analyses were carried out using SPSS version 20.0. Independent t-tests were used to compare the questionnaire scores between groups. Pearson’s correlations were calculated for some behavioural and self-reported outcomes (i.e. eating disorders symptoms, depression scores, frequencies of positive facial expressions, and attentional bias to sad faces). Repeated measures ANOVAs compared attentional bias to happy and sad facial expressions, ratings on the EAS and PANAS, and frequencies of positive and negative expressions and of looking away between participants groups (EDs vs. HCs).

The attentional bias scores to happiness and sadness were calculated following the analytical plan of MacLeod and Mathews [30]. The reaction times (RTs) for the trials when the probe replaces the emotional picture (sad or happy; valid trials) were subtracted from the RTs for the trials when the probe replaces the neutral picture (invalid trials; attentional bias score = invalid trials—valid trials). Data from trials with errors were discarded (3.9% for the ED group and 2.3% for HCs). Data were available for 132 participants (HCs = 69; EDs = 63; BN = 16).

The films were coded according to the Facial Expression Coding System (FACES [31]) adopting the methodology developed in our previous studies [15, 16]. The neutral film clip was used to obtain a baseline measure of mood and to eliminate the possible carry-over effects produced by the emotion-inducing video clips. Facial expressions during the neutral video clip were not coded. Expressions were categorised as a change to ‘positive’ or ‘negative’ facial display and coded for valence (positive or negative), intensity [1–4] and duration (seconds). Emotions were categorised as either positive or negative by following the FACES manual guide. An additional expression was considered if the initial facial expression did not return to a neutral expression or shifted to another affective facial display instead. The frequency the individual looked away from the screen was also counted, but the duration was not measured. Thus, the total scores derived from coding were: 1) frequency of positive expressions, mean intensity, mean duration; 2) frequency of negative expressions, mean intensity, mean duration; 3) frequency of looking away.

The FACES manual provides a clear description of the key features of facial expression. Two researchers from our in house team of raters who were blind to subject diagnosis and type of film cue (J.L. and C.R) made the ratings. The training they received included coding practice videotapes. Regular weekly meetings were used to calibrate the more difficult tapes with the supervisor (F.C.), who had had previous experience with the use of the FACES manual. The codings were independently undertaken on average 6 hours/week for 4 weeks. Practice and supervision continued until good agreement with standard ratings on our laboratory tapes was found. The overall inter-rater agreement (*κ* index) between the two coders (J.L., C.R.) reached an acceptable value of 0.79. This is similar to the inter-rater agreement we have obtained using this method in other studies from our laboratory [15, 16].

For the purpose of these studies, only congruent facial displays were included in the analysis (i.e. frequency of positive expression to happiness; and frequency of negative expression to sadness and anger). Following Davies and colleagues’ procedure [15], the frequency of facial expression was used as the prime index of emotional expression as frequency, intensity and duration were all significantly correlated (correlations conducted separately for each video clip; *p* < .0001). Technical difficulties with the film task (e.g. participant moves out of head shot) or participant refusing to have video tape made resulted in loss of some data points (data available for 109 participants: HCs = 66, EDs = 43).

All effect sizes (*ESs*) were calculated using Cohen’s *d* [32] and described as negligible (= 0 and < 0.15), small (≥ 0.15 and < 0.40), medium (≥ 0.40 and < 0.75), large (≥ 0.75 and < 1.10), very large (≥ 1.10 and < 1.45) and huge (> 1.45) [32].

## Results

### Socio-demographic and clinical variables

The demographic and clinical questionnaires were completed by the 116 participants (i.e. HCs = 71; AN = 33–19 with restrictive symptoms and 15 with binge/purge symptoms; BN = 12; table 1). Participants in the ED and HC groups did not differ significantly on age, but HCs had more years of education. Participants with AN had a significantly lower BMI than participants with BN and HCs. Overall, participants with an ED (AN or BN) showed significantly higher levels of eating disorders symptoms and depression, anxiety and stress than HCs on the EDE-Q and DASS questionnaires. In relation to social functioning, fewer participants in the ED group reported to be in a relationship than HCs.

### Attentional probe task: emotion processing (i.e. attentional bias to happiness and sadness)

**Fig 1:**
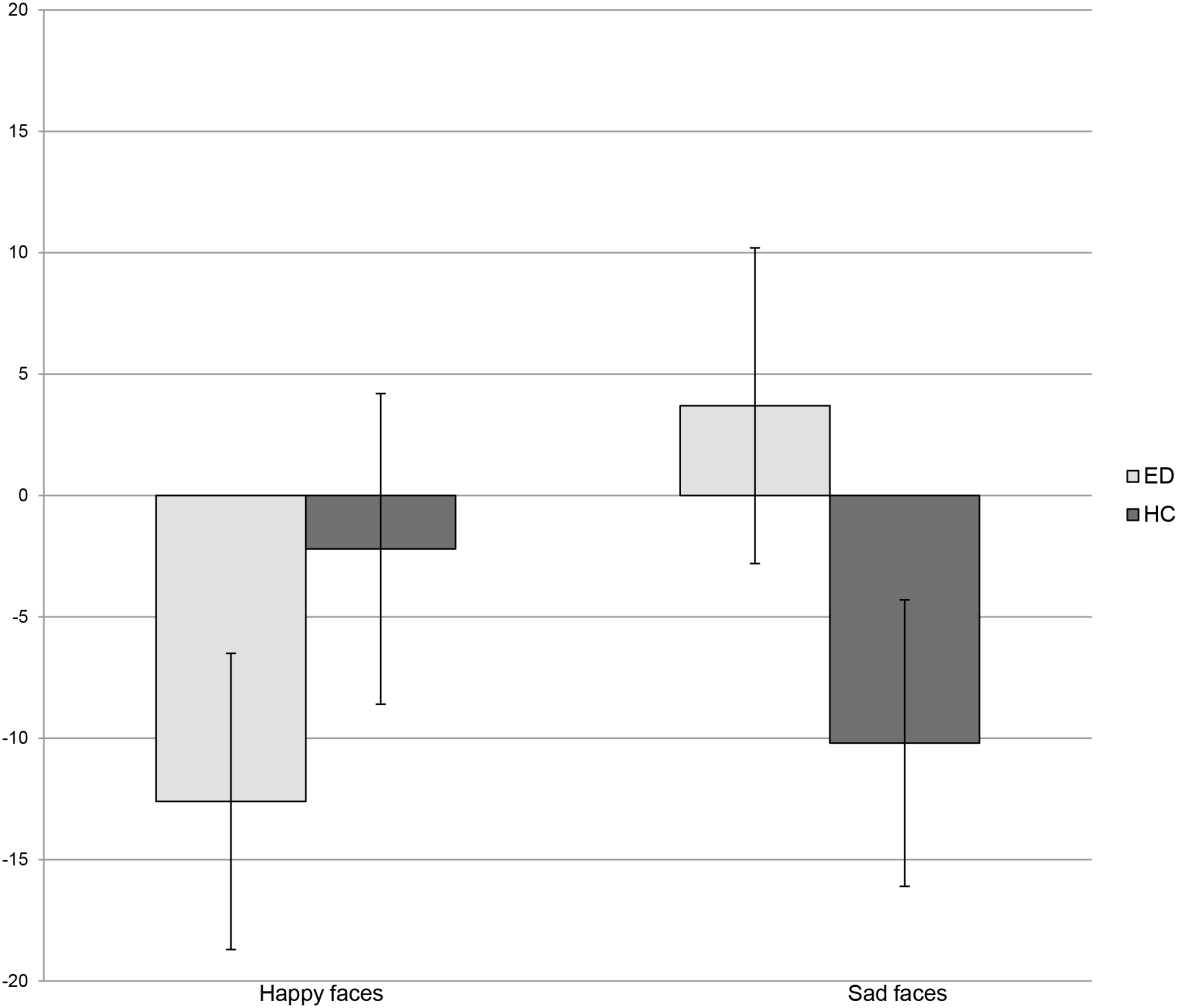
Attentional response to happy and sad expressions in participants with Eating Disorders and Healthy Controls. Attentional response to happy and sad facial expressions (500 ms) compared between currently ill people (EDs) and and healthy controls (HCs). Attentional bias scores expressed as means, in milliseconds (ms).

There was a trend towards an overall difference between groups in the attentional response to emotional cues (Group x Emotional Face: *F*_1, 130_ = 3.5; *p* = .06). Participants with an ED had a tendency for a stronger attentional disengagement from facial expressions of happiness than HCs (*t*_131_ = 1.4; *p* = .2). Also, mean scores showed that EDs had a bias towards sad expressions, whereas HCs disengaged from these stimuli (*t*_135_ = -1.5, *p* = .1). Fig 1 and Table 2 show the attentional response to happy and sad expressions in the ED and HC groups.

**Table 2:** Socio-emotional processes (attentional biases, emotion recognition, empathy and frequency of loss of attention and facial emotional expressions) evoked by video clips displaying happiness, sadness and anger. Scores are expressed as means and standard deviations for participants with eating disorders (EDs) and healthy controls (HCs). Effect sizes of differences are presented.

Process and sample size	Emotion valence	EDs	HCs	Effect size
Attentional bias (EDs = 63; HCs = 69)	Happiness	-12.6 (48.8)	-2.2. (52.3)	0.3
	Sadness	3.7 (52.3)	-10.2 (50)	0.1
Emotion recognition (EDs = 49; HCs = 72)	Happiness	12.8 (4.0)	13.9 (3.7)	0.28
	Sadness	11.3 (6.3)	12.5 (7.0)	0.17
	Anger	21.4 (9.2)	24.2 (7.2)	0.34
Emotional empathy (EDs = 49; HCs = 72)	Happiness	9.1 (8.2)	10.5 (6.3)	0.19
	Sadness	5.4 (5.4)	4.7 (5.1)	0.13
	Anger	9.2 (7.7)	9.2 (7.9)	0
Facial expressions* (EDs = 43; HCs = 66)	Positive to happiness	.5 (.9)	1.4 (1.2)	0.8
	Negative to sadness	.4 (.5)	.7 (.7)	0.47
	Negative to anger	.9 (.9)	1.5 (1.2)	0.54
Looking away* (EDs = 43; HCs = 66)	Happiness	.9 (1.7)	.2 (1.0)	0.53
	Sadness	1.6 (3.1)	1.0 (2.1)	0.2
	Anger	1.7 (3.9)	.3 (1.4)	0.52

Significant differences between groups are identified with a * next to the study’s outcomes.

### Video-clip task: emotion recognition

Participants with EDs and HCs reported similar congruent ratings of the three video clips (i.e. video clip displaying happiness rated as positive; video clips displaying sadness and anger rated as negative; Group *F*_1, 119_ = 3.0, *p* = .09). Similarly, no significant differences were observed between groups according to emotion rated (Group x Emotion: *F*_2, 238_ = 1.3, *p* = .3). Overall, the two groups reported significantly higher ratings for the video clip displaying anger (Mean = 23.1, SD = 8.1) than the video clips displaying happiness (Mean = 13.5. SD = 3.9; *t*_121_ = -15.9, *p* < .0001; *ES* = 1.5) and sadness (Mean = 12.0, SD = 6.7; *t*_120_ = 18.3, *p* < .0001; *ES* = 1.49].

### Video-clip task: empathy towards others

There were no overall differences between groups in the ratings of positive and negative emotions experienced during the video clips (Group *F*_1, 119_ = .06; *p* = .8). Participants with ED and HCs reported similar levels of negative emotions in response to the video clips displaying sadness and anger and similar levels of positive emotions in response to the video clip displaying happiness (Group x Emotion *F*_2, 238_ = 1.1, *p* = .3). Overall, the two groups reported significantly lower ratings for the video clip displaying sadness (Mean = 5, *SD* = 5.3) than the video clips displaying happiness (Mean = 9.9, *SD* = 7.1; *t*_120_ = 7.5, *p* < .0001; *ES* = 0.78) and anger (Mean = 9.2, *SD* = 7.7; *t*_120_ = 7.8, *p* < .0001; *ES* = 0.63).

### Video-clip task: facial expressions to positive and negative emotions

**Fig 2:**
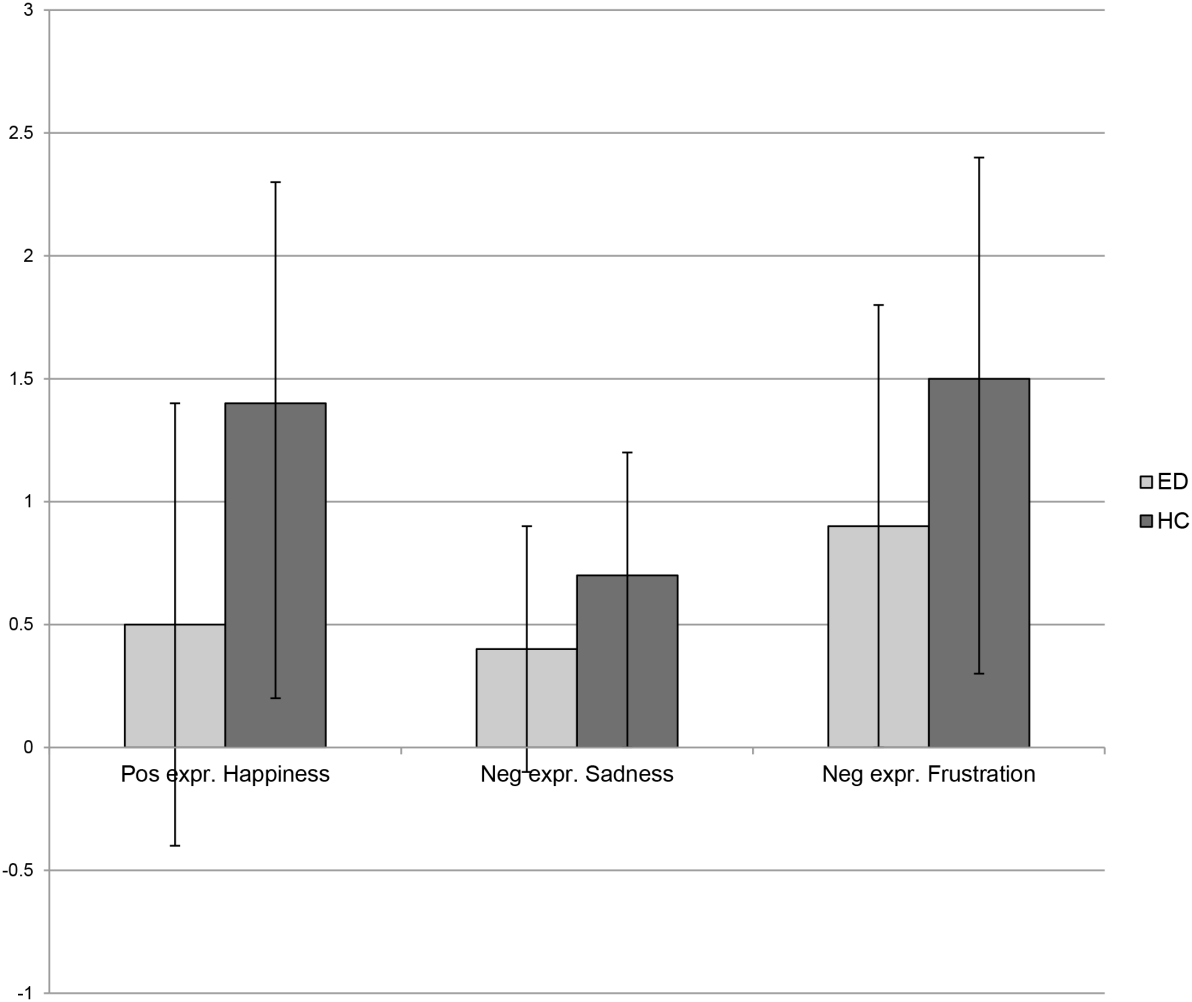
Frequencies of positive and negative facial expressions shown by participants with Eating Disorders and Healthy Controls. Mean frequencies of positive facial expressions shown in response to happy displays and negative facial expressions shown in response to negative displays (i.e. sadness and anger) in participants with Eating Disorders (EDs) and Healthy Controls (HCs).

Overall, participants with EDs displayed fewer emotional expressions than HCs (Group: *F*_1, 107_ = 18.2, *p* < .0001). In particular they showed significantly fewer positive expressions in response to the video clip displaying happiness (*t*_111_ = 3.5, *p* = .001) and fewer negative expressions in response to the video clips displaying anger (*t*_110_ = 2.4, *p* = .02) and sadness (*t*_109_ = 2.3, *p* = .02). Frequencies of facial expressions are shown in Fig 2.

EDs and HCs had a different pattern of facial responsivity to specific emotions (Group x Emotion: *F*_2, 214_ = 3.0, *p* = .05). Participants with an ED showed significantly more facial expressions in response to the video clip displaying anger than happiness (*t*_43_ = -2.1, *p* = .04; *ES* = 0.44) and sadness (*t*_43_ = 4.3, *p* < .0001; *ES* = 0.68), whereas no significant differences were observed in the number of facial expressions to happiness and sadness (*t*_42_ = 1.0, *p* = .3; *ES* = 0.13). Healthy controls showed significantly more facial expressions in response to happiness and anger than sadness (happiness vs. sadness: *t*_65_ = 4.1, *p* < .0001; *ES* = 0.71; anger vs. sadness: *t*_66_ = 6.6, *p* < .0001; *ES* = 0.81; happiness vs. anger: *t*_65_ = -.5, *p* = .6; *ES* = 0.08).

### Film task: frequency of looking away

Overall, participants with EDs looked away more frequently than HCs during the video clips (Group: *F*_1, 107_ = 5.4, *p* = .02), across all of the emotional conditions (Group x Emotion: *F*_2, 214_ = 2.6, *p* = .09).

Supplementary analyses showed that participants with AN or BN did not differ on any of the measures considered.

### Correlations between behavioural measures and eating disorders symptoms and depression scores

Across the entire sample of participants, the number of positive expressions produced were negatively correlated with the level of depression (*r*_101_ = -.3, *p* = .007) and eating disorders symptoms (*r*_101_ = -.2, *p* = .01).

## Discussion

The aim of this study was to experimentally investigate specific processes of social-cognition in patients with EDs. In particular, the perception and recognition of others’ emotional expressions, empathy (i.e. own emotional response to others’ emotional expressions) and evoked facial emotional expressions were explored. Patients were similar to healthy controls in recognising and subjectively responding to others’ emotions. However, they showed less facial expressivity and attention to the video-clips of prototypical emotional displays. There was also a trend suggestive of an attentional bias towards sad faces and away from happy faces in the attentional probe task.

Although the recognition of and subjective empathic response to others’ emotional displays were similar to those observed in controls, the evoked facial emotional expressions were lower in patients. This is consistent with the findings from studies which have examined more complex aspects of socio-emotional functioning. For example, a lack of evoked facial emotional response to tragic and comic film clips has been found in both adults [15] and adolescents with EDs [16]. Moreover, patients display less facial emotional response when playing a therapeutic video game [33].

Lack of facial affect is one of the key negative features described in schizophrenia [34] and is associated with negative prognosis. Several studies have used different methodologies to capture this symptom in this condition [34–39]. Further investigations have identified this feature also in participants with depression [35, 40] and autism [36, 41]. Interpersonal matching of non-verbal expression during social processes is important throughout development and critical for social functioning, empathy and understanding of another person’s state of mind [42]. In EDs, the lack of positive facial expressions in response to happiness may be associated with social anhedonia [43] and the lack of response to negative emotional displays could be part of a maladaptive emotional regulation strategy based on suppression [44] and emotional avoidance [45, 46]. This is supported by the finding that patients turned away from the video-clips more often than HCs, as observed also in previous studies conducted in both adults [15] and adolescents [16]. These anomalies in social communication may contribute to the high level of stress seen in carers. Individuals who suppress their facial expression of affect are regarded as less amiable by others who also react to this behaviour with increased physiological arousal [47].

The failure to find a moderate sized difference in attention to happy faces is surprising. In a previous study using the attentional probe task with faces depicting warmth and compassion rather than happiness, we found a moderate sized disengagement from these stimuli in patients with EDs [9]. These findings suggest that there is a degree of specificity in the attentional response to different types of facial positive expressions. Interestingly, healthy controls also did not have a bias towards happy faces. This replicates the findings of a similar study conducted in a sample of Korean women [48]. In his seminal work on emotional expression, Ekman described many different types of smiles [49] and distinguished the Duchenne smile, which communicates positive emotions, from other types of smiles, which may be aversive and processed as a threat by some individuals [50].

The small, non-significant bias towards sad faces again suggests some degree of specificity in the profile of patients’ social emotional response as a moderate sized attentional bias has been found with negative stimuli representing critical hostile faces [9]. It is possible that the attentional probe task and timing of pictures display were not sensitive enough to powerfully demonstrate these effects to sadness. Other possible explanations are that the critical rejecting faces were more salient (i.e. threatening) than sad expressions or that patients do not respond to basic discrete emotions in a biased way.

### Strengths and Limitations

The strengths of this study are: 1) the investigation of several distinct processes and constructs of social cognition in patients with AN or BN; 2) the inclusion of behavioural, as well as self-report measures; 3) the use of basic discrete emotions. Nevertheless there are also some limitations. Missing data for some of the questionnaires and recordings of facial expressions might have limited the power of the study. There was a large variance in the attentional bias scores and this might be due to the version of the attentional probe task we used (i.e. vertical vs. horizontal dots as target stimuli) or sample heterogeneity. There is some evidence for less variance being associated with the use of letters as targets, or with the identification of the position of the stimuli, rather than their discrimination [51]. The experimenter remained in the room whilst participants completed the attentional probe task and this might have affected their responses to the emotional pictures. The self-report measures assessing the emotional response to the video clips may have not been sensitive enough to detect differences between samples. The detection of physiological, as well as psychological responses to the stimuli might provide additional information on the differences between samples. The video-clips were presented following a fixed order and a neutral video clip was used to neutralise potential carry over effects. The use of a randomised order of presentation could have been more appropriate to control for carry over effects. Although we had high measures of inter-rater reliability for the facial expressions coding it is possible that a more sophisticated rating measure may have reduced the variance. We used novel video clips to depict discrete emotional expressions and validated them only in a sample of healthy controls. Also, it is possible that confounding features in these clips, such as gender and age of the subject, may have affected the emotional response of the participants.

### Clinical implications

There is interest in developing interventions for EDs which target problems in social processing. For example, treatments including education about the function of emotions and the benefits of emotional awareness, such as Emotion Acceptance Behaviour Therapy (EABT) and Radically Open-Dialectical Behaviour Therapy (RO-DBT) have been piloted in the treatment of AN [52, 53]. The Maudsley Model of Anorexia Nervosa Treatment for Adults (MANTRA) also contains a module relating to overcoming emotional avoidance [54]. These treatments involve a "top down" approach to emotional regulation, by teaching new skills of cognitive and emotional control.

These results might also have implications for training of carers. It is possible that the lack of affect makes it difficult for them to have empathy with the suffering and anxiety that individuals with EDs endure.

## Conclusion

In this study, participants with EDs did not show difficulties in recognising and appropriately responding to others’ basic discrete emotions when using self-report measures. On the other hand, they displayed less evoked facial expression and avoidance of the emotional displays. There were small, non significant differences in the attentional processing of sad and happy faces.

Despite severe social impairment, patients might experience only dissociable and specific problems in social cognition, perhaps resulting from downstream effects of other symptoms (e.g. nutritional imbalance), rather than primary deficits. These anomalies may contribute to the problems in social emotional functioning seen in EDs.
